# Adding value to myocardial perfusion SPECT/CT studies that include coronary calcium CT: Detection of incidental pulmonary arterial dilatation

**DOI:** 10.1097/MD.0000000000011359

**Published:** 2018-08-10

**Authors:** Charles Marcus, Prasanna Santhanam, Matthew J. Kruse, Mehrbod S. Javadi, Lilja B. Solnes, Steven P. Rowe

**Affiliations:** The Russell H. Morgan Department of Radiology and Radiological Science, Johns Hopkins University School of Medicine, Baltimore, MD.

**Keywords:** myocardial perfusion, myocardial SPECT/CT, pulmonary artery dilatation, pulmonary hypertension

## Abstract

The aim of the present study was to evaluate the incidence of undiagnosed pulmonary arterial dilatation using the gated computed tomography (CT) images acquired in patients with an otherwise normal ^99m^Tc-sestamibi single-photon-emission CT (SPECT)/CT myocardial perfusion study.

This was a retrospective review of 200 consecutive patients (100 men, mean age 58.7 years) who underwent a myocardial perfusion ^99m^Tc-sestamibi SPECT/CT study with normal perfusion and with gated CT images acquired for coronary calcium scoring. The CT images were reviewed using a previously validated mean main pulmonary artery diameter (mPAD) measurement method which has been correlated with pulmonary arterial hypertension (PAH). Clinical information on multiple comorbidities was also retrieved. Previously reported mPAD cutoffs (>29.5 and >31.5 mm) were used to stratify patients.

Indications for the study included dyspnea on exertion (58.9%), preoperative workup (22.3%), and chest pain (13.9%). The mean mPAD measurement was 26.3 mm (±0.5). There was a significant correlation between body mass index and mPAD (correlation coefficient [ρ]: 0.28; *P* < .001). About 23% (46/200) of patients had mPAD > 29.5 mm and 15.0% (30/200) of patients had mPAD > 31.5 mm. From previous work, these cutoffs have a sensitivity and specificity for PAH of 70.8%, 79.4% and 52.0%, 90.2%, respectively. Among patients undergoing a preoperative myocardial perfusion study, 35.6% (16/45) patients had mPAD > 29.5 mm and 26.7% (12/45) patients had mPAD > 31.5 mm. There was a higher prevalence of congestive heart failure (62.5% vs 19.6%; *P* < .001) and hypertension (78.3% vs 21.7%; *P* < .02) in patients with mPAD > 29.5 mm. Similarly, there was a high prevalence of congestive heart failure (*P* < .001), hyperlipidemia (*P* < .04), and hypertension (*P* < .04) in patients with mPAD > 31.5 mm.

Incidental pulmonary arterial dilatation (mPAD ≥ 29.5 mm) can be detected in a large number of patients with normal myocardial perfusion scintigraphy and correlates with multiple different comorbidities. The mPAD can be measured in all patients undergoing gated imaging as part of a myocardial perfusion study, and PAH may be considered as an alternative explanation for symptoms in some patients without perfusion deficits. The data to make this potential diagnosis is already being acquired and represents an opportunity to add value to the interpretations of otherwise negative myocardial perfusion studies.

## 1 Introduction

Heart disease remains the number 1 cause of death in the United States. According to the National Hospital Ambulatory Medical Care Survey in 2013, the number of visits with a principal hospital discharge diagnosis of ischemic heart disease after an emergency department visit was 267,000 and with a diagnosis of heart disease excluding ischemic causes was 748,000.^[1]^ The number of cardiac stress tests ordered at office visits accounted to 5.0 million in the United States.

The ^99m^Tc-sestamibi myocardial perfusion single photon emission computed tomography/computed tomography (SPECT/CT) is an important and commonly employed noninvasive diagnostic test to evaluate for myocardial perfusion. The aim of diagnosing myocardial perfusion deficits is to identify functionally significant coronary artery disease (CAD) in symptomatic patients and to assign appropriate risk stratification.^[2]^ Myocardial perfusion SPECT/CT (MPS) imaging is also used for screening for underlying CAD in asymptomatic and at-risk patients undergoing a preoperative cardiovascular evaluation. The SPECT component of the myocardial perfusion imaging is commonly performed with gating to provide information on left ventricular volumes at rest and after stress, left ventricular ejection fraction, and regional wall motion.^[3]^

Incidental findings are part of the routine radiologic reporting workflow and describe findings which are not primarily the focus of the clinical context of the study.^[4–8]^ The clinical implications of such findings can be widely variable and a categorization of incidental findings according to clinical relevance is important. Documentation and communication of clinically relevant incidental findings are essential parts of the radiologic reporting process. The aim of this study was to evaluate for incidental pulmonary artery (PA) dilatation (a noninvasive measurement that is a surrogate for pulmonary arterial hypertension [PAH]) in patients undergoing MPS studies with a resultant normal myocardial perfusion since PAH and CAD can have overlapping nonspecific symptoms such as exertion-induced shortness of breath, fatigue, weakness, angina, and syncope, and to also evaluate the presence of comorbidities that may correlate with the presence of PAH. The presentation may be modified by diseases that cause, or are associated with, PAH as well as other concurrent diseases.^[9]^ PAH is a debilitating disease and appropriate treatment after early diagnosis is crucial for satisfactory patient outcomes.^[10]^ Studies have shown that CT can be useful in the evaluation and management of patients with pulmonary hypertension. CT findings suggestive of pulmonary hypertension include dilated PA, increased main PA/abdominal aorta ratio, PA calcification, tortuosity of the PA, rapid tapering of the PA, and mosaic attenuation of the lung parenchyma.^[11]^ For accurate correlation, the PA measurements from the gated-CT acquired routinely in MPS examinations for coronary calcium scoring were stratified based on a previously validated, large study correlating PA measurements on CT with right heart catheterization pulmonary arterial pressure (PAP) measurements. PA measurement cutoffs of 29.5 and 31.5 mm were used in this study and these cutoffs have demonstrated a sensitivity and specificity of 70.8%, 79.4% and 52.0%, 90.2%, respectively.^[12]^

## 2 Materials and methods

### 2.1 Patient selection

We retrospectively reviewed 200 consecutive patients who had undergone an MPS study with a resultant normal myocardial perfusion and with a gated coronary calcium scoring CT from October 26, 2015 to March 31, 2016 at our institution. This is the same cohort of patients previously described in another manuscript with a separate hypothesis and different findings and conclusions.^[13]^ The study was approved by the Institutional Review Board under a waiver of informed consent in accordance with HIPAA guidelines. Studies with abnormal myocardial perfusion or studies performed without gated CT were excluded. Patients included in the study did not have an established diagnosis of PAH prior to the study. The indication for performing each of the MPS studies was retrospectively determined using clinical notes and the MPS requisition forms in the medical records. The patient demographic data, past medical history, smoking and/or alcohol use history, and type of stress study (exercise vs pharmacologic) were recorded.

### 2.2 Image acquisition

Gated SPECT perfusion images were acquired over a 180° arc with a dual-head SPECT system (Siemens Medical Systems, Erlangen, Germany) equipped with a low-energy, high-resolution, parallel-hole collimator 90 to 120 minutes after injection of ^99m^Tc-sestamibi (30 mCi) at stress. SPECT imaging was performed for 15 minutes with 32 views, at 25 seconds per view, using 64 × 64 matrix size with a zoom factor of 1.45. The cardiac cycle was divided into 8 equal intervals with a 20% window centered over the 140 keV photo peak of ^99m^Tc. Gated transaxial images were reconstructed using the Butterworth-filtered back-projection method (order 5; cutoff frequency 0.40) and displayed as short-axis as well as horizontal and vertical long-axis slices. Attenuation-correction CT acquisition was started immediately after the emission scan was completed and then a separate calcium scoring CT was obtained. All scans were taken in patients in the supine position with breath holding at full inspiration. The following acquisition parameters were used: slice thickness 2.4 mm, effective mA of 75, 130 kVp, 0.42 seconds rotation time, and a pitch of 0.9. CT scans were performed without iodinated contrast. For the attenuation correction CT, slice thickness of 5.0 mm with a scan time of 0.35 seconds was used. Rest ^99m^Tc-sestamibi SPECT/CT images are not routinely acquired at our institution after a normal stress perfusion acquisition.

### 2.3 Image analysis

**Figure 1:**
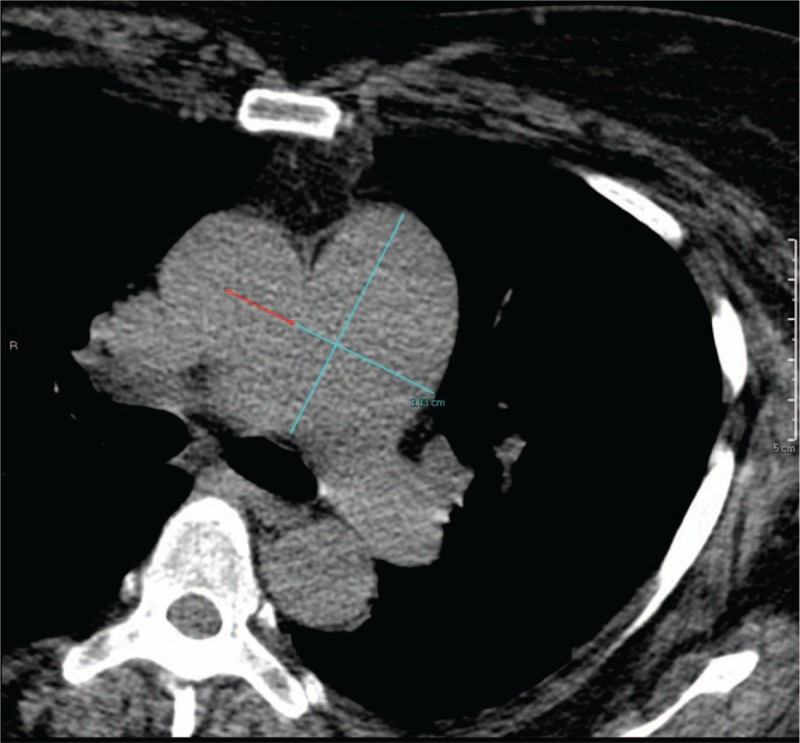
Measurement of main pulmonary artery diameter (mPAD). Axial gated-computed tomography (CT) image demonstrating measurement method for determining mPAD. The mPAD is measured along the line that originates from the center of the ascending thoracic aorta and passes perpendicular to the long axis of the main pulmonary artery, at the level of the pulmonary artery bifurcation.

All MPS images were electronically retrieved on a workstation. An experienced nuclear medicine reader reviewed the gated CT images in axial planes. A validated mean main PA diameter (mPAD) measurement (in keeping with the previously described method demonstrated by Mahammedi et al that has been previously correlated with PAP obtained by right heart catheterization) was used to measure the mPAD and stratify patients. The work by Mahammedi et al had demonstrated that the mPAD and mPAD/ascending aorta diameter (AAD) ratio were found to have the highest correlation with PAPs (*r* = 0.51 and 0.53, respectively; *P* < .001). For the sake of convenience, the mPAD was used in this study. A threshold of mPAD >29.5 and >31.5 mm used by the authors demonstrated sensitivity and specificity of 70.8%, 79.4% and 52.0%, 90.2%, respectively. The authors also showed that there was a statistically significant difference (*P* < .0001) in mPAD and mPAD/AAD ratio between controls and patients with pulmonary hypertension. Among the mPAD measurement methods proposed by the authors, the method with the highest correlation coefficient with PAP (*r* = 0.51; *P* < .0001) was used to measure the mPAD. The mPAD by this method was estimated along a line drawn from the center of the aorta such that it passes perpendicular to the axis of the main PA, when the main PA has a straight appearance at the level of the PA bifurcation on an axial section^[12]^ (Fig. 1).

### 2.4 Statistical analysis

We used mPAD cutoffs of 29.5 and 31.5 mm as detailed earlier. Age, body mass index (BMI) (in kg/m^2^), coronary calcium score, and mPAD were the continuous variables while gender, race, indication for the stress test, comorbidities such as smoking history, known CAD, hypertension, congestive heart failure (CHF), kidney disease, and diabetes were treated as categorical variables. We tabulated the frequency distribution of the different variables among different categories of patients based on the measured mPAD. We performed correlation between BMI and mPAD and age and mPAD. The comparison for the presence of comorbidities between the different categories of patients was done using Chi-squared tests. Finally, we performed a multivariate logistic regression analysis to examine the relationship between the different variables with an increased mPAD based on the cutoffs described above as the dependent variable.

## 3 Results

### 3.1 Patient characteristics

We identified 200 consecutive patients who had undergone an MPS study with a resultant normal myocardial perfusion. Of the 200 patients, 100 were men and 100 were women. The mean age (values are in mean ± standard deviation) was 58.7 (±10.4) years. Table 1 summarizes the patient demographics, BMI, symptoms at presentation, past medical history, smoking and/or alcohol use, coronary calcium score, primary indication of the scan, and comorbidities.

**Table 1:**
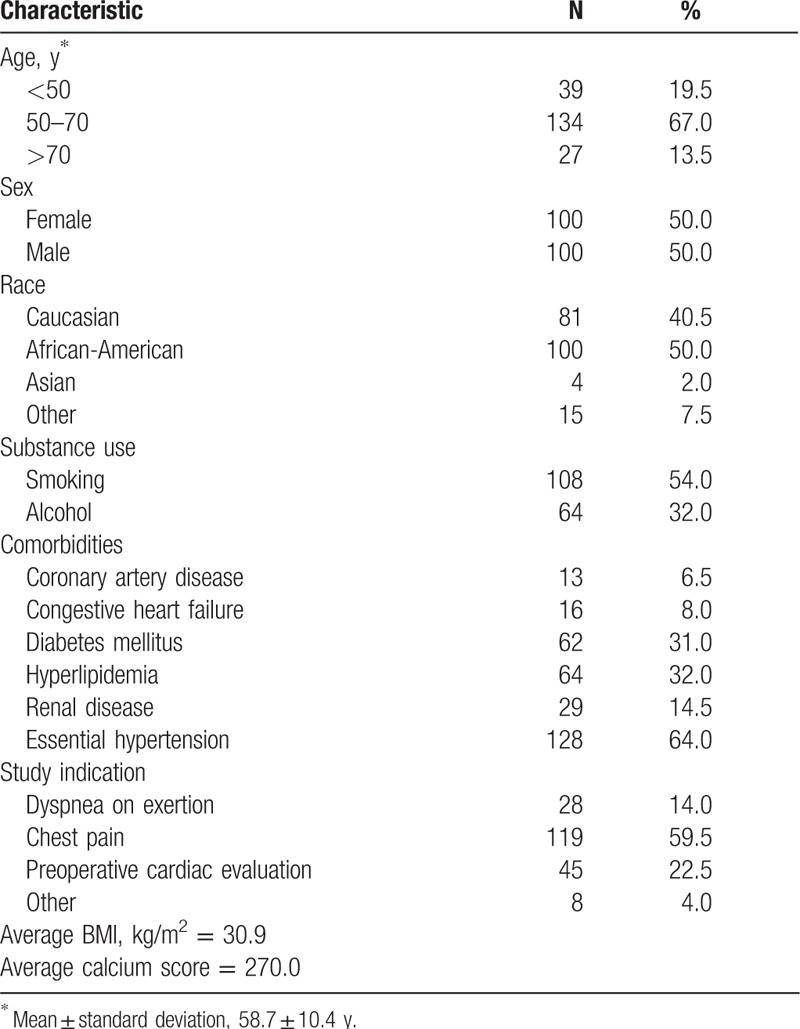
Characteristics of the 200 patients included in the study.

### 3.2 Categorization of the MPS studies and mPAD measurement

**Figure 2:**
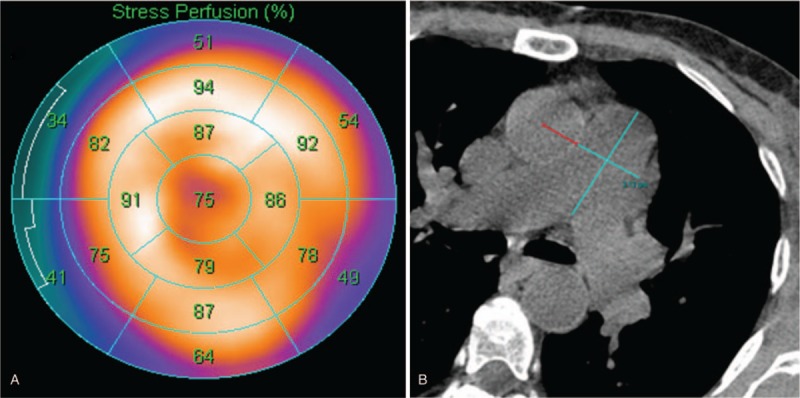
^99m^Tc sestamibi stress myocardial perfusion map (A) and axial gated-computed tomography (CT) image (B) of the main pulmonary artery of a 60-year-old hypertensive woman who underwent a ^99m^Tc sestamibi myocardial perfusion scintigraphy as part of a preoperative cardiac evaluation for a liver transplantation. The myocardial perfusion map during stress demonstrates normal myocardial perfusion with no regions of decreased radiotracer distribution. The axial gated-CT image demonstrates a dilated main pulmonary artery, measuring 31.3 mm.

Of the 200 studies, the most common indication was dyspnea on exertion (58.9%), followed by preoperative workup (22.3%) and chest pain (13.9%). The mean mPAD measurement was 26.3 mm (±0.5). In our cohort of 200 patients with normal myocardial perfusion, 23.0% (46/200) of patients had mPAD of ≥29.5 mm. About 15.0% (30/200) patients had mPAD ≥ 31.5 mm. Among patients undergoing preoperative cardiac evaluation, 37.8% (17/45) had mPAD > 29.5 mm and 28.9% (13/45) had mPAD > 31.5 mm. A case example is illustrated in Figure 2.

There was a significant correlation between BMI and mPAD (Spearman ρ = 0.277, *P* < .001), and no correlation between mPAD and age (*P* = .85). About 59.7% of patients with mPAD < 29.5 mm had hypertension as compared to 78.3% in patients with mPAD ≥ 29.5 mm (*P* = .02). There was an increased prevalence of CHF in patients with mPAD ≥ 29.5 mm (21.7% vs 3.9%, *P* < .01). There was no difference in the prevalence of smoking, diabetes, hyperlipidemia, or renal disease between the groups. The prevalence of comorbidities in these 2 groups of patients has been described in Table 2. About 50% of patients with CHF had mPAD ≥ 31.5 mm, compared to 12% without CHF (*P* < .01).

**Table 2:**
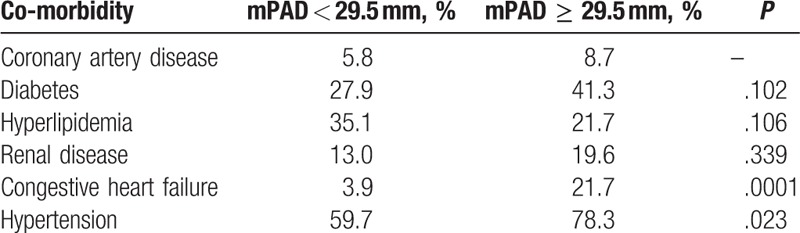
Prevalence of comorbidities in patients with main pulmonary artery diameter (mPAD) < 29.5 mm and mPAD ≥ 29.5 mm.

The logistic regression model was statistically significant (χ^2^(7) = 32.7, *P* < .01). The model explained 25.8% (Nagelkerke *R*^*2*^) of the variance and correctly classified 81.0% of cases. Of the predictor variables, those that were statistically significant were: age, BMI, and CHF, though the effect was smaller with age and BMI. In the multivariate analysis involving age, BMI, smoking, diabetes, hypertension, CAD and CHF, persons with CHF were 1.8 times more likely to have mPAD ≥ 29.5 mm.

## 4 Discussion

The MPS is a commonly performed diagnostic study to evaluate for coronary perfusion abnormalities in symptomatic patients and as part of the preoperative workup in patients undergoing some surgical procedures. Evaluation of myocardial perfusion is crucial, considering the debilitating outcomes. Prompt and accurate diagnosis of compromised myocardial perfusion is crucial and has been the focus of upcoming research to improve the performance of diagnostic imaging.^[14,15]^ As mentioned earlier, symptoms of CAD and pulmonary hypertension often overlap, making the clinical differentiation of these entities challenging. Left undiagnosed and untreated, pulmonary hypertension may have a debilitating effect on the lives of patients; studies have shown that the symptoms of pulmonary hypertension can adversely affect the physical, emotional, and social factors governing patient health-related quality of life.^[10]^

Our study has shown that 23.0% patients in this cohort had mPAD > 29.5 mm and 15.0% patients had mPAD > 31.5 mm, measured from the gated-CT images acquired routinely as part of the myocardial perfusion scintigraphy imaging protocol. These cutoffs were used based on previously validated work by Mahammedi et al who correlated the mPAD cutoffs with the gold standard PAP measured by right heart catheterization in 298 patients with known pulmonary hypertension and in 102 controls, and is one of the seminal series on correlations between chest CT and hemodynamic measurements of PAP. The diagnostic performance of this method has been described earlier in the paper and the cutoff of 29.5 mm yielded the highest diagnostic accuracy. The 31.5 mm threshold yielded the highest specificity. Since the number of patients in our study population with mPAD > 31.5 mm was low (n = 30), the 29.5 mm threshold was used for our statistical correlation.^[12]^ Previous studies have shown that obesity is an independent risk factor for PAH. Similarly studies have shown that PAH is a common complication of left heart dysfunction, which is often encountered in patients with advanced age, essential hypertension, atrial fibrillation, diabetes mellitus, etc.^[16,17]^ In keeping with these findings, our study showed that there was a significant correlation between BMI and mPAD. Our findings also demonstrate that there was a higher incidence of hypertension and CHF in patients with mPAD > 29.5 mm. Similarly there was a higher incidence of CHF in patients with mPAD > 31.5 mm. An interesting observation was that in patients with mPAD > 29.5 mm with probable pulmonary hypertension, only 22.0% had known CHF and 9.0% had known CAD. Common overlapping symptoms in patients with pulmonary hypertension and CAD are dyspnea and chest discomfort. This suggests that these symptoms, in the context of normal myocardial perfusion, may be attributed to pulmonary hypertension and this should be considered as an alternative diagnosis that can often be evaluated for on the same imaging examination.

The second most common indication for MPS was preoperative cardiac evaluation prior to elective major surgical procedures such as organ transplantation. Studies have shown that PAH is a frequent complication of portal hypertension and is frequently diagnosed in patients with end-stage renal disease who are undergoing dialysis. PAH has been found to negatively impact outcome after renal and liver transplants.^[18]^ Among patients undergoing preoperative cardiac evaluation in our study, more than one-third of patients (37.8%) had mPAD > 29.5 mm and 28.9% had mPAD > 31.5 mm, which is a significant percent of patients and should provide valuable information to the treating physician and anesthesiologist to plan appropriately for intraoperative and postoperative care and treatment.

The limitations in this study include its retrospective nature and the use of a surrogate imaging finding (ie, mPAD) for the detection of PAH without corroborative right heart catheterization pressure measurements. A longer-term prospective study that includes recommendations in the clinical reads of the MPS studies based on mPAD measurements and follow-up of patients would clarify the preliminary findings in this study.

In the era of maximizing cost-effective diagnostic imaging in the evaluation and management of patients with a special focus on cost- and time-effective, noninvasive evaluation of CAD, any added information that can be derived from already available images at no additional cost will benefit patients and health providers in early diagnosis and appropriate etiology evaluation and management of patients.^[19]^ Since early diagnosis and management is crucial in PAH for reducing disease-specific morbidity and mortality, commenting on the presence of PA dilatation, which can be easily assessed from the gated-CT images acquired as part of MPS, should be an important aspect of the dictated reports of these examinations. Even in those patients presenting for preoperative evaluation or other symptoms that would not normally be mistaken for underlying CAD, the presence of possible PAH is an important potential comorbidity to be noted by the interpreting imaging specialist and considered by the referring clinician.

## 5 Conclusion

The PA dilatation can be seen in a high percentage of patients undergoing MPS with a resultant normal myocardial perfusion. The PAD can be measured using an easily reproducible method from the gated-CT images acquired as part of the MPS study at no-additional cost or time to the patient and reporting the PAD should be considered in all MPS studies as it may indicate the presence of PAH, an important potential comorbidity.

## Author contributions

**Conceptualization:** Charles Marcus, Matthew Kruse, Mehrbod Javadi, Lilja Solnes, Steven Rowe.

**Data curation:** Charles Marcus, Prasanna Santhanam, Mehrbod Javadi, Lilja Solnes, Steven Rowe.

**Formal analysis:** Charles Marcus, Prasanna Santhanam, Matthew Kruse, Mehrbod Javadi, Lilja Solnes, Steven Rowe.

**Investigation:** Charles Marcus, Prasanna Santhanam, Matthew Kruse, Mehrbod Javadi, Steven Rowe.

**Methodology:** Charles Marcus, Prasanna Santhanam, Mehrbod Javadi, Lilja Solnes, Steven Rowe.

**Project administration:** Steven Rowe.

**Resources:** Steven Rowe.

**Supervision:** Mehrbod Javadi, Lilja Solnes, Steven Rowe.

**Writing – original draft:** Charles Marcus, Prasanna Santhanam, Matthew Kruse, Mehrbod Javadi, Lilja Solnes, Steven Rowe.

**Writing – review & editing:** Charles Marcus, Prasanna Santhanam, Matthew Kruse, Mehrbod Javadi, Lilja Solnes, Steven Rowe.

## References

[1] National Center for Health Statistics: Heart Disease. CDC/National Center for Health Statistics; 2017. Available at https://www.cdc.gov/nchs/fastats/heart-disease.htm. Accessed June 22, 2017.

[2] Hedgire SS Osborne M Verdini DJ Updates on stress imaging testing and myocardial viability with advanced imaging modalities. Curr Treat Options Cardiovasc Med 2017;19:26. 28316034 10.1007/s11936-017-0525-7 PMC5842358

[3] Miller TD Askew JW Anavekar NS Noninvasive stress testing for coronary artery disease. Heart Fail Clin 2016;12:65–82. 26567975 10.1016/j.hfc.2015.08.006

[4] Hoang JK Langer JE Middleton WD Managing incidental thyroid nodules detected on imaging: white paper of the ACR Incidental Thyroid Findings Committee. J Am Coll Radiol 2015;12:143–50. 25456025 10.1016/j.jacr.2014.09.038

[5] Megibow AJ Baker ME Morgan DE Management of incidental pancreatic cysts: a white paper of the ACR Incidental Findings Committee. J Am Coll Radiol 2018;15:591. 10.1016/j.jacr.2017.11.016 29483051

[6] Sebastian S Araujo C Neitlich JD Managing incidental findings on abdominal and pelvic CT and MRI, part 4: white paper of the ACR Incidental Findings Committee II on gallbladder and biliary findings. J Am Coll Radiol 2013;10:953–6. 24295947 10.1016/j.jacr.2013.05.022

[7] Heller MT Harisinghani M Neitlich JD Managing incidental findings on abdominal and pelvic CT and MRI, part 3: white paper of the ACR Incidental Findings Committee II on splenic and nodal findings. J Am Coll Radiol 2013;10:833–9. 24183552 10.1016/j.jacr.2013.05.020

[8] Berland LL Overview of white papers of the ACR Incidental Findings Committee II on adnexal, vascular, splenic, nodal, gallbladder, and biliary findings. J Am Coll Radiol 2013;10:672–4. 23816427 10.1016/j.jacr.2013.05.012

[9] “2015 ESC/ERS Guidelines for the diagnosis and treatment of pulmonary hypertension. The Joint Task Force for the Diagnosis and Treatment of Pulmonary Hypertension of the European Society of Cardiology (ESC) and the European Respiratory Society (ERS).” Nazzareno Galie, Marc Humbert, Jean-Luc Vachiery, Simon Gibbs, Irene Lang, Adam Torbicki, Gerald Simonneau, Andrew Peacock, Anton Vonk Noordegraaf, Maurice Beghetti, Ardeschir Ghofrani, Miguel Angel Gomez Sanchez, Georg Hansmann, Walter Klepetko, Patrizio Lancellotti, Marco Matucci, Theresa McDonagh, Luc A. Pierard, Pedro T. Trindade, Maurizio Zompatori and Marius Hoeper. Eur Respir J 2015; 46: 903-975. Eur Respir J 2015;46:1855–6. 26621899 10.1183/13993003.51032-2015

[10] Delcroix M Howard L Pulmonary arterial hypertension: the burden of disease and impact on quality of life. Eur Respir Rev 2015;24:621–9. 26621976 10.1183/16000617.0063-2015 PMC9487616

[11] Freed BH Collins JD Francois CJ MR and CT imaging for the evaluation of pulmonary hypertension. JACC Cardiovasc Imaging 2016;9:715–32. 27282439 10.1016/j.jcmg.2015.12.015 PMC4905589

[12] Mahammedi A Oshmyansky A Hassoun PM Pulmonary artery measurements in pulmonary hypertension: the role of computed tomography. J Thorac Imaging 2013;28:96–103. 23096163 10.1097/RTI.0b013e318271c2eb

[13] Santhanam P Marcus C Solnes LB Incidental pulmonary arterial dilatation and coronary calcifications in patients with hypertension and normal findings on myocardial perfusion technetium-99m sestamibi single-photon emission computed tomography. J Clin Hypertens (Greenwich) 2017;19:1054–5. 28646514 10.1111/jch.13043 PMC8031330

[14] Gao Z Lie X Qi Song Automatic segmentation of coronary tree in CT angiography images. Int J Adapt Control Signal Process 2017;1–9.

[15] Zhen X Zhang H Islam A Direct and simultaneous estimation of cardiac four chamber volumes by multioutput sparse regression. Med Image Anal 2017;36:184–96. 27940226 10.1016/j.media.2016.11.008

[16] Mehta S Vachiery JL Pulmonary hypertension: the importance of correctly diagnosing the cause. Eur Respir Rev 2016;25:372–80. 27903659 10.1183/16000617.0104-2016 PMC9487545

[17] Barnett CF Alvarez P Park MH Pulmonary arterial hypertension: diagnosis and treatment. Cardiol Clin 2016;34:375–89. 27443135 10.1016/j.ccl.2016.04.006

[18] Frost AE The intersection of pulmonary hypertension and solid organ transplantation. Methodist Debakey Cardiovasc J 2016;12:10–3. 10.14797/mdcj-12-4s1-10 PMC5347179 28298957

[19] Zeb I Abbas N Nasir K Coronary computed tomography as a cost-effective test strategy for coronary artery disease assessment - a systematic review. Atherosclerosis 2014;234:426–35. 24769305 10.1016/j.atherosclerosis.2014.02.011

